# The Relationship Between Internal Employer Branding and Talent Retention: A Theoretical Investigation for the Development of a Conceptual Framework

**DOI:** 10.3389/fpsyg.2022.859614

**Published:** 2022-03-18

**Authors:** Rizwan Raheem Ahmed, Muhammad Azam, Jawaid Ahmed Qureshi, Alharthi Rami Hashem E, Vishnu Parmar, Nor Zafar Md Salleh

**Affiliations:** ^1^Faculty of Management Sciences, Indus University, Karachi, Pakistan; ^2^Faculty of Management Sciences, Shaheed Zulfikar Ali Bhutto Institute of Science and Technology, Karachi, Pakistan; ^3^Department of Financial and Administrative Sciences, Ranyah University College, Taif University, Taif, Saudi Arabia; ^4^Institute of Business Administration, University of Sindh, Jamshoro, Pakistan; ^5^Azman Hashim International Business School, Universiti Teknologi Malaysia, Skudai, Malaysia

**Keywords:** employer brand image, employer branding, talent retention, internal branding, employees’ engagement, job design

## Abstract

The focus of this paper is to develop a comprehensive conceptual framework for the relationship between internal employer brand image and talent retention. An extensive and semi-systematic literature review identified a number of antecedents and consequences that have been empirically tested in various cutting-edge research studies that were conducted around the world. The existing literature is reviewed using a topical approach, and 66 research studies, most recent from various repositories, were carefully chosen and reviewed based on the criteria. Such studies have been discerned and synthesized in order to establish a complete and accurate understanding of the phenomenon. Finally, a comprehensive and relatively rich conceptual framework has been proposed for future empirical explanations in various social settings *via* prospective research.

## Introduction

A brand is a specific symbol, emblem, name, character, or insignia that represents, signifies, or characterizes a specific product (Kotler and Armstrong, 2017). Brand’s etymology may be traced back to the classic German language, where it means “fire.” The term “brand” means “burning” in ancient English. The brand image corresponds to the perceived quality, features, characteristics, qualities, benefits, and side effects in the minds of present or prospective customers (Keller and Kotler, 2015; Biswas and Suar, 2016). Employer brand image refers to the perceptual impression associated to an employer’s qualities and attributes in the minds of existing employees (internal customers) and potential employees (Barrow and Mosley, 2007; Priyadarshi, 2011). Dowling (1993) and Keller (1993) demonstrated that the employer image converts into the tangible benefits or the asset of the organization.

Employer branding has been introduced from the marketing discipline to the realm of human resource management (HRM) (Stokes, 2015; Stokes et al., 2016; Valle, 2016; Meyer and Xin, 2017; Theurer et al., 2018; Deepa and Baral, 2019; Purusottama, 2019). Employer branding has two dimensions: the first is a source of attraction for new employees and the second is a source of motivation for incumbents to stay with the employer in the long run (Theurer et al., 2018; Deepa and Baral, 2019). As a result, both prospective candidates and current employees are stakeholders in employer branding.

The difficulty and concern of a successful chief executive officer (CEO) is the recruitment and retention of the talented staff at all ranks (Meyer and Xin, 2017). This challenge is being met with novel techniques developed by combining HRM and marketing strategies. Presently, talent and talent segmentation are prominent HRM subjects that are, in fact, applications of marketing concepts, such as customer and customer segmentation. The top management has learned from business experience curves that long-term retention of talented workers is a basic necessity for developing a competitive advantage over others (Meyer and Xin, 2017).

External employer branding refers to the focus of employer branding strategies on prospective employees, while internal employer branding refers to the focus on incumbents. This research study adds to the theories of employer branding and work design. It is largely concerned with internal employer branding, which is divided into antecedents and repercussions. Antecedents are variables that contribute to the development of an employer’s brand image, whereas repercussions are created by the employer’s brand image. Both sides of the nexus are described in logical sequence in this article.

### Antecedents of Internal Employer Branding

Chhabra and Sharma (2014) examined organizational culture, brand name, and compensation as antecedents of employer branding. Jain and Bhatt (2015) arrived at the conclusion that, in order to attract potential employees and retain current employees, it is critical to provide them with organizational support and infrastructure during their early days in the organization; additionally, organizational culture is equally important for achieving the best results from the previous two determinants. Organizational culture and organizational identity are two factors that contribute to an employer’s positive reputation and attraction (Jiang and Iles, 2011; Sageder et al., 2018). As per understanding of internal or external antecedents driven from the studies of Saraswathy and Balakrishnan (2017) and Davies et al. (2018), organizational culture and organizational identity are internal antecedents of employer brand image. In advanced economies, potential applicants have a wide selection of potential employers to choose from, so they make their decision to apply for a position extremely carefully, taking into account a variety of variables, such as trustworthiness (Beck and Kenning, 2015; Davies et al., 2018; Sageder et al., 2018), prestige (Davies et al., 2018), organizational culture, and organizational identity (Jiang and Iles, 2011; Sageder et al., 2018).

Employer branding encompasses organizational culture, interpersonal relations, systems, and organizational values (Singh and Rokade, 2014; Sharma and Prasad, 2018). A pleasant, hygienic, and conducive environment makes employees comfortable at work, which eventually may lead to their satisfaction (Singh and Rokade, 2014). Employees love to stay longer with employers who are providing work-life balance and competitive compensation (Singh and Rokade, 2014; Tanwar and Prasad, 2016). Carless and Imber (2007) and Carpentier et al. (2017) highlighted that the attractiveness of the employer is critical in catching the attention of prospective employees looking for work. Carpentier et al. (2017) have explored six factors that influence employer branding, which include pay, advancement, task diversity, atmosphere, meaningfulness, and work-life balance.

Immediate surroundings, such as work settings, people, furniture, equipment, building, and infrastructure etcetera are called working environments (Dessler, 2016). Jain and Bhatt (2015) have taken infrastructure solely as one determinant of employer branding. Employees prefer to work where people are collaborative, mutual respect is assured, the latest technology is available, work settings are safer and hygienic (Barrow and Mosley, 2007; Priyadarshi, 2011). Sharma and Prasad (2018) identified five constituents of employer branding, which include compensation and benefits (Chhabra and Sharma, 2014; Carpentier et al., 2017), work-life balance, work environment, brand strength, and organizational culture.

Carpentier et al. (2017) and Sharma and Prasad (2018) have taken work-life balance as another antecedent of employer brand image. Srivastava and Bhatnagar (2010), Jiang and Iles (2011), Chhabra and Sharma (2014), Beck and Kenning (2015), Jain and Bhatt (2015), Carpentier et al. (2017), Saraswathy and Balakrishnan (2017), Urbancova et al. (2017), Astrachan et al. (2018), Kaur et al. (2018), and Sageder et al. (2018) have taken work environment and organizational culture as predictors for employees’ attraction in the employer. Martin et al. (2016), Urde and Greyser (2016) and Astrachan et al. (2018) have taken brand strength as one of the predictors to employer brand image. Kaur et al. (2018) claimed that organizational culture is critical in attracting internal customers, namely, the personnel. Organizational culture represents the philosophy of the organization and the mindset of management (Srivastava and Bhatnagar, 2010). Truss et al. (2012) have endorsed that training and development is one important factor that creates a positive brand image of the employer.

Training and development are viewed from two perspectives: the employers and the employees. As a result, it must be used effectively because it benefits both sides. Employers want their employees to be updated, competitive, and ready to meet the threats and challenges; due to which, they provide training and manage development programs for their growth in knowledge, skills, and abilities (KSAs). Employees, on the other hand, seek training to supplement their KSAs and to participate in various development programs at work in order to raise their market value (Minchington, 2010; Truss et al., 2012; Dessler, 2016).

Earlier studies of Bhatnagar (2007) and Vaiman (2008) provide sound support to the arguments of Truss et al. (2012) and Dessler (2016) that training/learning and development programs and career planning are core factors that affect employer brand Image. Employees always appreciate opportunities to learn new things at work and overall people development; however, sincerity in building efficient career progression plans has been a tough demand by employees. As a result, these factors are vital in making employees happy at work, which ultimately generates and enhances a positive employer brand image. Astrachan et al. (2018) have taken four components of employer branding which include identity, image, reputation, and brand. Authors described that today’s employees, apart from good remuneration, look for their and their work’s identity in the organization. Astrachan et al. (2018) have taken the term image as the perceived image of the employer in the market that how people are mentally associated with the organization. In other words, how an employer wants to influence its offerings in the job market (Camara, 2011). Astrachan et al. (2018) have taken reputation from Urde and Greyser (2016) that people’s opinions, views, judgments, and mental association with the employer following employer’s actions in long term. Astrachan et al. (2018) have also taken the concept of the brand from Urde and Greyser (2016) that brand is a collection of value backed by promise and their assured delivery. Astrachan et al. (2018) concluded that these four components collectively form employer branding in the jobs market.

Hackman and Oldham (1975) and Oldham et al. (2005) have addressed their very own theory of work design, namely, the Job Characteristics Model, which is used to measure the attitude of employees toward the job. Authors have theorized and advocated that employees who are on the higher side of the Motivating Potential Score (MPS) have a positive attitude toward and image of their employer, which causes them to become engrossed in their work (job engagement), and they have a higher probability of performing well and remaining associated with their employer in the long run. Flexibility is an implicit agreement or willingness to change according to the situation or circumstances by either party, i.e., employer or employee (Berkman et al., 2010; Dessler, 2016; Urbancova et al., 2017). Flexibility includes options for working hours, such as morning, evening, or night shifts, driving to the office or working from a virtual office, formal or relaxed attire, and so on. Flexibility is essential for putting employees at comfort and allowing them to operate without stress (Dessler, 2016; Urbancova et al., 2017). The literature matrix containing a tabular summary of antecedents of employer branding is depicted in Table 1.

**TABLE 1:** Antecedents of employer branding.

Authors	Dimensions/factors
Astrachan et al. (2018)	Organizational culture, identity, image, reputation, brand
Barrow and Mosley (2007)	
Barrow and Mosley (2007)	Collaboration, learning and development, latest technology, mutual respect, work settings
Beck and Kenning (2015)	Trustworthiness
Berkman et al. (2010)	Flexibility
Bhatnagar (2007)	Career planning
Camara (2011)	Image
Carless and Imber (2007)	Attractiveness
Carpentier et al. (2017)	Advancement, attractiveness, pay, task diversity, meaningfulness, atmosphere, work-life balance
Chhabra and Sharma (2014)	Compensation, organizational culture, brand name
Davies (2008)	Trustworthiness, prestige
Deepa and Baral (2019)	Development value, marketing practices, economic value, social value, work value, organizational prestige
Dessler (2016)	Training and development, flexibility
Jain and Bhatt (2015)	Organizational culture, organizational support, infrastructure
Jiang and Iles (2011)	Organizational environment, organizational culture
Kaur et al. (2018)	Organizational culture
Martin et al. (2016)	Brand propositions, effective communication of brand propositions, brand loyalty
Minchington (2010)	Training and development
Oldham et al. (2005)	Task identity
Priyadarshi (2011)	Collaboration, training and development mutual respect, latest technology, work settings
Purusottama (2019)	Marketing practices
Sageder et al. (2018)	Trustworthiness
Saraswathy and Balakrishnan (2017)	Trustworthiness, prestige
Sharma and Prasad (2018)	Compensation and benefits, organizational culture, brand strength, work environment, work-life balance,
Singh and Rokade (2014)	Competitive compensation, organizational culture, organizational values, system, interpersonal relations, work-life balance
Srivastava and Bhatnagar (2010)	Organizational culture
Stokes (2015)	Marketing practices, culture, values, transparency
Tanwar and Prasad (2016)	Competitive compensation, work-life balance
Truss et al. (2012)	Training and development
Urbancova et al. (2017)	Flexibility
Urde and Greyser (2016)	Reputation, brand
Vaiman (2008)	Training and development
Valle (2016)	Clear message of brand propositions, meaningful brand propositions

### Consequences of Internal Employer Branding

Kahn (1990) theoretically explained the phenomenon of employees’ engagement at work as harnessing self, while performing the job emotionally, cognitively, and physically. The author developed three characteristics of employees’ engagement, i.e., meaningfulness, availability, and safety by which a specific employee establishes engagement with their work. Fredrickson (2000) provided another perspective on the phenomenon of employees’ engagement as positive emotions, which broaden the thought process and span of favorable actions at the workplace, which benefits employers and employees in return. The author asserted that such emotions help in building new ideas, reaching creative solutions, and turning colleagues into friends etcetera.

The emotional and cognitive harnessing asserted by Kahn (1990) has convergence with positive emotions asserted by Fredrickson (2000) because emotions either positive or negative are based upon one’s cognitive understanding of the particular situation, event, or individual. Therefore, the studies of Kahn (1990) and Fredrickson (2000) are sharing definitional commonalities. Leiter and Maslach (1998) and Maslach and Leiter (2008), considered employees’ engagement as opposite behavior to the employees’ burnout. They concluded in their seminal study that an employee cannot be engaged at work if he or she is suffering from predictors of burnout behavior, such as stress, a non-conducive atmosphere, toxic boss conduct, and unrealistic deadlines.

Contrary to the studies of Leiter and Maslach (1998), another study by Schaufeli and Bakker (2004) refers to engagement as an attitude, but not behavior. Schaufeli and Bakker (2004) asserted that employees’ engagement is feelings of being positive and having work-oriented mindset, which is backed by absorption, dedication, and vigor in wholesome. The authors defined absorption as a state of intense attention during work in which time moves too rapidly and it is impossible to disconnect from the task at hand. Dedication is defined as feelings of being excited for, inspired by, honored to be at work, and viewing work as a challenge that helps employees stay happy and optimistic about their work. Vigor is defined as the height of an employee’s energy level and the flexibility that an individual exhibits at work, which assists employees in dealing with weariness at work.

Lockwood (2006) asserted that post-industrial progress, where work nature is intangible and performance is based on originality, efficiency, and flexibility, demands high employees’ engagement, which is one of the main challenges that employers are facing. Bhatnagar (2007) reflected that providing employees with engrossing and conducive environment leads to earning two-pronged benefits, i.e., enhanced brand equity of employer and retention in longer run. Bhatnagar (2007) reflected that employees’ engagement has been the second most important HRM issue, while retention of talented employees being first, for the last 10 years. In this context, Bhatnagar (2007) asserted that effective managerial practices demonstrate the employer’s concern toward human capital, which causes employees to be engaged and decrease in the rate of turnover. Eventually, such managerial concern and employees’ response create a significant impact on individual performance and the overall performance of the organization.

Warr and Inceoglu (2012) explained the relationship of personality traits of the big five model, i.e., extroversion, agreeableness, conscientiousness, emotional stability, and openness to experience with the construct of employees’ engagement. Authors found the relationship of employees’ engagement in the short run as a function of the long-term big five traits explicitly emotional stability and two sub-factors socially proactive and achievement-oriented from two core factors, extroversion and conscientiousness, respectively. Vorina et al. (2017) have explained employees’ engagement as behavior, but not attitude in a broader spectrum while testing the relationship of its main antecedents and the impact of employees’ engagement on different work-related behaviors. Vorina et al. (2017) have explained the significant relationship of employees’ engagement with its four antecedents, which include credible leadership, supportive co-workers, job and career satisfaction, and high-performing organizations. Vorina et al. (2017) have also testified the relationship of employees’ engagement with its descendent behaviors and outcomes, which include satisfied and loyal customers, productive and profitable organizations, high-performing workforce, and committed employees.

The study of Meere (as cited in Wang, 2016) concluded that employees’ engagement has at least three stages, i.e., actively disengaged, not engaged, and engaged. Wang (2016) summarized “actively disengaged” employees who are not satisfied, involved in complaints, and they spread negative energy among colleagues; “not Engaged” employees who lack enthusiasm and positive energy, and working for serving the time only; “engaged” employees who are profoundly connected, dedicated, and passionate at the workplace. Okolo et al. (2018) and Kamel (2019) have taken the job characteristics model (Hackman and Oldham, 1975) into account to explain its relationship with employees’ engagement and talent retention. It has been stated that organizations that prioritize talent acquisition and retention create appealing and stimulating work designs and conducive work environments for their employees. Akhir et al. (2018) have explained the relationship of employees’ engagement with its leading consequence, i.e., turnover intention. Turnover intention is viewed as the inverse of employee retention, which makes sense given that leaving or planning to leave the organization is a projected attitude of incumbents, who would behave differently if employee retention is viewed as a result of employee engagement.

Perceived corporate social responsibility (Perceived CSR) also causes employees’ engagement with the mediation effect of employees’ empathy (Tian and Robertson, 2019). Madu et al. (2019) have taken aesthetic workplace as a cause of employees’ engagement. The aesthetic workplace, on the other hand, refers to an organization that is highly beautiful in its physical structure, ambience, and internal appeal, all of which are sub-components of the organizational environment (Carpentier et al., 2017; Astrachan et al., 2018; Davies et al., 2018). Dut̨u and Butucescu (2019) have taken employees’ engagement as a consequence caused by transformational leadership, since such leaders possess the phenomenal power to motivate, delight, and engage the sub-ordinates in a way that become heavily engaged with the firm and perform far more than the expected targets. Conclusively, meaningfulness, availability, and safety (Kahn, 1990), absorption, dedication, and vigor (Schaufeli and Bakker, 2004), and emotional stability, socially proactive, achievement-oriented (Inceoglu and Warr, 2012) are dimensions to measure the employees’ engagement; actively disengaged, not engaged, and engaged (Wang, 2016) are types of employees’ engagement, while, positive emotions (Fredrickson, 2000), autonomy, participative decision-making, and mutual trust (Lockwood, 2006), conducive environment (Bhatnagar, 2007), credible leadership, supportive co-workers, job and career satisfaction, and high-performing organizations (Vorina et al., 2017) are antecedents or predictors of employees’ engagement.

The phenomenon of talent retention is associated with employees’ employment tenure with a particular employer (Dessler, 2016). After an employee is hired, the next milestone is to utilize their talent in the long run. There are at least two reasons behind this interest. The first is that replacing an employee with a new hire is always more expensive than retaining an existing employee (Dessler, 2016). Second, a high frequency of recruiting people for a certain position entails a variety of non-monetary costs, such as orientation, training, and development, and normal performance during the initial months of hire, the possibility of theft of business or organizational secrets by employees who have left the workplace, and so forth.

Arasanmi and Krishna (2019) have explained the relationship of employer branding with employees’ retention. Authors have empirically proved the triangular relationship among perceived organizational support, organizational commitment, and employees’ intentions. West et al. (2019) have empirically explained the relationship of employees’ retention with one of the antecedents of employer branding, i.e., compensation and benefits through mediation effect of job satisfaction. Qureshi (2019) has taken high-performance work systems into account to empirically explain their relationship with employees’ commitment and employees’ retention at will. Empirical findings have shown that in the Gulf countries of the Middle East, organizations with high-performing work systems have more devoted employees who want to stay with the business in the long run. In the presence of a just and rigorous performance management culture, the most significant aspects of such high-performing work systems have been training, development, and compensation and benefits. Sukriket (2014) has testified and empirically proved the inverse relationship of employees’ recognition, communication, behavior of co-workers, benefits from employer, job conditions at the workplace, nature of work assigned, operating procedures for working, supervision by seniors, pay structure, and promotion in a career with turnover intentions.

Rubenstein et al. (2018) have carried out a comprehensive meta-analysis of employees’ turnover intention and categorized nine antecedents that include individual attributes, aspects of job, traditional job attitudes, newer personal conditions, organizational context, person-context interface, external job market, attitudinal withdrawal, and employee behavior. Syed et al. (2018) have explained the relationship of turnover intentions with the work-life imbalance and job stresses. Nie et al. (2018) have explained the theoretical relationship of work-family integration and equal career opportunity with turnover intentions with moderating role of supervisor’s gender. Conclusively, the turnover intention is the reverse to the construct of intention to stay and antecedents of the earlier can be taken as antecedents of the latter in the inverse relationship. The literature matrix containing tabular summary of consequences of Employer Branding is demonstrated in Table 2:

**TABLE 2:** Consequences of employer branding.

Author(s)	Dimensions/Factors
**Employees’ engagement**	
Schaufeli and Bakker (2004)	Absorption, dedication, vigor
Inceoglu and Warr (2012)	Achievement oriented, emotional stability, socially proactive
Wang (2016)	Actively disengaged, engaged, not engaged
Madu et al. (2019)	Aesthetic workplace
Lockwood (2006)	Autonomy/freedom, mutual trust, participative decision making
Kahn (1990)	Availability, meaningfulness, safety
Bhatnagar (2007)	Conducive environment
Vorina et al. (2017)	Credible leadership, supportive co-workers. high performing organizations, job and career satisfaction
Tian and Robertson (2019)	CSR activities, employees empathy
Kamel (2019)	Job design
Fredrickson (2000)	Positive emotions
Dut̨u and Butucescu (2019)	Transformational leadership
**Intention to stay**	
Rubenstein et al. (2018)	Aspects of jobs, attitudinal withdrawal, employees’ behavior, external job market, individual attributes, newer personal conditions, organizational context, traditional job attitude
Sukriket (2014)	Behavior of co-workers, benefits from employer, communication, employees’ recognition, job conditions, nature of work, operating procedure, pay structure, person context interface, promotions, supervision
Nie et al. (2018)	Career opportunities, work-life balance
West et al. (2019)	Compensation and benefits
Qureshi (2019)	Compensation and benefits, employees’ commitment, performance management, recruitment and selection, training and development
Syed et al. (2018)	Job stress, work-life balance
Arasanmi and Krishna (2019)	Organizational commitment, perceived organizational support

## Materials and Methods

We fetched literature pertaining to core and sub-themes of Employer Branding from the digital library of the Higher Education Commission (HEC) of Pakistan. We accessed more than 200 research articles out of which 66 articles qualified the criteria of high *recency* and *relevance*. The topical approach is opted for searching the articles from repositories to conduct the semi-systematic literature review. Selected research publications are most recent except some classical state-of-the-art articles and examined in connection to the relevant themes with the goal of designing a robust conceptual framework. Review-driven findings are segregated based on their themes and sub-themes; followed by clubbing of related themes to form more abstract themes for increasing parsimony of conceptual framework. The semi-systematic literature review overcomes the hurdles of a systematic review and synthesizes the vital notions, theories, frameworks, and developments (Snyder, 2019; Bhat et al., 2020). An extensive literature analysis identified a nexus of Employer Branding, which is difficult to understand without its deconstruction; thus, the following paragraphs discuss relationship-based disintegration, which aids in understanding the framework’s overall image. Positive and negative notions represent the right and inverse relationship between two variables, respectively.

### Literature Gap

The phenomenon of employer branding (Barrow and Mosley, 2007; Priyadarshi, 2011; Carpentier et al., 2017; Saraswathy and Balakrishnan, 2017; Urbancova et al., 2017; Astrachan et al., 2018; Kaur et al., 2018; Sageder et al., 2018; Sharma and Prasad, 2018; Deepa and Baral, 2019) along with its antecedents and its consequence employees’ engagement (Kahn, 1990; Schaufeli et al., 2002; Inceoglu and Warr, 2012; Warr and Inceoglu, 2012; Wang, 2016; Vorina et al., 2017), talent retention at will (Arasanmi and Krishna, 2019; Qureshi, 2019; West et al., 2019) has been explored and explained through different research studies but intensive literature review lacks empirical shreds of evidence of theoretical relationship among all three constructs, i.e., employer branding, employee’s engagement, and talent retention in one particular social setting, which means theoretical gap exists in the literature.

Job design (Okolo et al., 2018; Kamel, 2019) has been explained as an antecedent of employees’ engagement and talent retention, but literature review lacks sound evidence where it has been considered as an antecedent of employer brand image and having mediation effect on it, i.e., employer brand image with two consequences, e.g., employees’ engagement and talent retention. Therefore, a theoretical gap exists in the extant literature.

Since the emergence of cultural dimensions back in 1981, Hofstede (2011) explored nine cultural dimensions on the basis of which one social setting is different from others. Without testing in the specific social setting, research studies and phenomena testified in other social settings might not bring the same results. The triangular relationship of employer branding, employees’ engagement, and employees’ talent retention needs to be testified in different social settings before generalizing and practicing.

### Selection of Research Papers and Flow of Ideas

**FIGURE 1:**
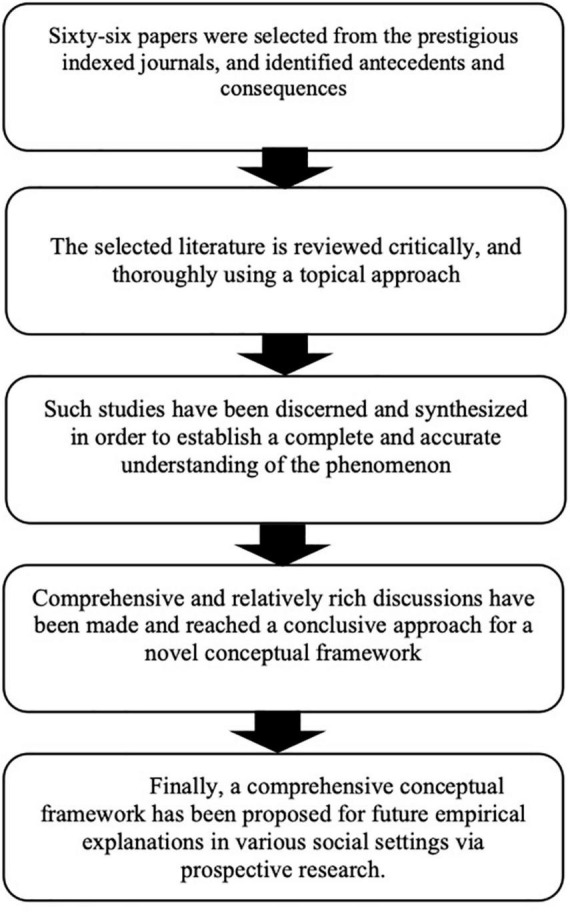
Flow of ideas for novel conceptual framework.

The papers were selected from reputed indexing, such as Web of Science, Scopus, ABDC, and ABS, on the basis of different dimensions or factors which are demonstrated in Table 1 (antecedents of employer branding) and Table 2 (consequences of employer branding). The details of selected research articles are also provided in Tables 1, 2. The flow of ideas for the construction of a novel conceptual framework as cascaded in Figure 1.

## Discussion for Final Conceptual Framework

### The Proposed Framework

The semi-systematic literature review is undertaken using a topical approach, and 66 research publications most recent from various repositories have been selected and examined in connection to the relevant variables with the goal of designing a conceptual framework based on theoretical underpinning. The semi-systematic literature review overcomes the hurdles of a systematic review and synthesizes the vital notions, theories, frameworks, and developments (Snyder, 2019; Bhat et al., 2020). An extensive literature analysis identified a nexus of Employer Branding, which is difficult to understand without its deconstruction; thus, the following paragraphs discuss relationship-based disintegration, which aids in understanding the framework’s overall image.

### Competitive Compensation

Perceived competitive compensation has been of high relevance with internal employer branding as it is explained in many recent past research studies (e.g., Chhabra and Sharma, 2014; Singh and Rokade, 2014; Sukriket, 2014; Tanwar and Prasad, 2016; Carpentier et al., 2017; Sharma and Prasad, 2018; Deepa and Baral, 2019). It is quite likely that in a particular social situation, employees who receive pay from their employers that is quite competitive to market rates have identified their employers with a positive brand image and are more likely to stay linked with the business in the long run. It has been described as the top or the second most important component with a high impact on employer branding and employee retention in previous studies noted above. As a result, this concept has been adopted as one of the primary antecedents of employer branding.

### Organizational Environment

The perceived organizational environment has been taken into account and explained in recent past research studies (e.g., Jiang and Iles, 2011; Priyadarshi, 2011; Chhabra and Sharma, 2014; Singh and Rokade, 2014; Beck and Kenning, 2015; Jain and Bhatt, 2015; Stokes, 2015; Tanwar and Prasad, 2016; Carpentier et al., 2017; Saraswathy and Balakrishnan, 2017; Urbancova et al., 2017; Astrachan et al., 2018; Davies et al., 2018; Kaur et al., 2018; Sageder et al., 2018; Sharma and Prasad, 2018; Deepa and Baral, 2019) as an antecedent of internal employer branding. The organizational environment itself is an abstract concept, which is explained through culture, work setting, interpersonal relationships, organizational justice, and work-life balance etcetera. Employees in a particular social context who have a suitable work environment, rich culture, good interpersonal interactions, fairness and justice, and a healthy work-life balance are more likely to have a positive picture of the employer brand and to stay linked with the employer in the long run. As a result, this concept has been adopted as one of the primary antecedents of employer branding.

### Career Progression

Perceived career progression has been taken into account and explained in recent past research studies (e.g., Minchington, 2010; Priyadarshi, 2011; Truss et al., 2012; Davies et al., 2018; Deepa and Baral, 2019) as an antecedent of internal employer branding. Career progression is explained through learning opportunities, training investment orientation, career growth opportunities, succession planning, and so on. Employees who have opportunities to learn, managers with a positive attitude toward training and development, organizations concerned with employees’ career development, and organizations that practice succession planning at work are more likely to have a positive image of the employer brand and to remain associated with the employer in the long run. As a result, this concept has been adopted as one of the primary antecedents of employer branding.

### Employer Prestige

Perceived Reputation of Employer in the market has been taken into account and explained in recent past research studies (e.g., Minchington, 2010; Priyadarshi, 2011; Truss et al., 2012; Davies et al., 2018; Deepa and Baral, 2019; Lu et al., 2020) as an antecedent of internal employer branding. The employer’s reputation in the market is explained by the image of the employer viewed by people outside the firm, excellence in market competition, perceived CSR, and the like. It is highly anticipated that in a given social setting, employees who perceive that their employer has a prestigious image among people outside the organization, have established healthy competition with excellence among competitors and have engaged in effective CSR toward society, are more likely to have a rich image of the employer brand and are more likely to remain associated with employer in the long run. As a result, this concept has been adopted as one of the primary antecedents of employer branding.

### Job Design

Job design has been taken into account and explained in research studies (e.g., Okolo et al., 2018; Kamel, 2019) as a core antecedent of employees’ engagement. Job design is explained through skills variety, task identity, task significance, autonomy, and feedback of job characteristics model (Hackman and Oldham, 1975) that is perceived on the basis of experiences of the incumbents. An employee with a better perceived work design is more likely to become involved with the task. Despite having motivational features, the literature lacks evidence that work design is a precursor to employer branding. As a result, this concept has been adopted as an antecedent for both employee engagement and employer branding (Ashraf et al., 2018).

### Employees’ Engagement

Employees’ engagement has been taken into account and explained in recent past research studies (e.g., Inceoglu and Warr, 2012; Wang, 2016; Vorina et al., 2017; Akhir et al., 2018; Dut̨u and Butucescu, 2019; Madu et al., 2019; Tian and Robertson, 2019) as an antecedent and consequence of different constructs. According to the literature review, most of the time, employee engagement is viewed as a result of various positive antecedents, such as high-performing organizations, supportive work colleagues, job satisfaction, career growth opportunities, socially proactive workplace, more conducive environment, and so on. Critical synthesis aids comprehension of the fact that the aforementioned antecedents of employee engagement are essentially antecedents and factors of employer branding; hence, employer branding is one of the main antecedents of employees’ engagement. Employee engagement, on the other hand, has been identified as a predictor of a variety of outcomes, such as perceived performance, employee retention, and employee turnover intention. As a result, it has been discovered that the construct of employees’ engagement is both an antecedent and a consequence of different constructs; in fact, it serves as a mediator between its antecedents and consequences.

### Talent Retention

Recent studies have taken into account and explained talent retention (Arasanmi and Krishna, 2019; Qureshi, 2019; West et al., 2019), employer brand image, and employee engagement as a result of employer branding strategies. On the other hand, numerous researchers have explained its counterpart construct, turnover intention (e.g., Sukriket, 2014; Nie et al., 2018; Rubenstein et al., 2018; Syed et al., 2018). As a result of various constructs that emerged during the literature review, such as recognition of employees’ work, communication with colleagues, colleague behavior, job conditions, nature of the work, supervision, pay structure, promotion-related policies and practices, individual attributes of employees, work-life balance, and economic situation, and so forth. These indications can also be used to predict talent retention at will, although the link between said indicators and talent retention will be inverse, as it is with turnover intentions. Critical syntheses of these precursors result in similarities to antecedents of employer branding. Gilmore (2017) developed the process of antecedents leading to behavior and then to outcomes employees’ engagement is taken into account in this research study as a behavior with several antecedents that comprise the construct of employer branding; once employees is engaged with their work, they will have a consequence in the form of willful employees’ retention.

### Final Conceptual Framework

**FIGURE 2:**
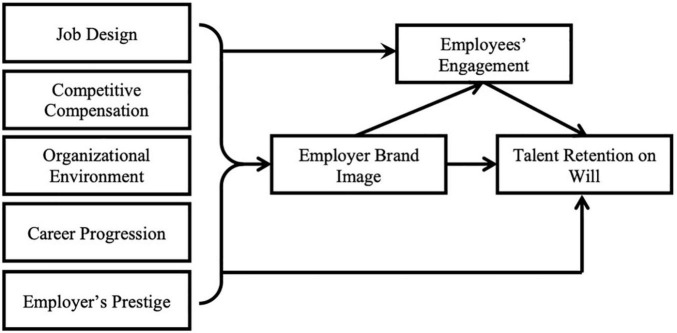
The final conceptual framework (Minchington, 2010; Jiang and Iles, 2011; Priyadarshi, 2011; Inceoglu and Warr, 2012; Truss et al., 2012; Chhabra and Sharma, 2014; Singh and Rokade, 2014; Sukriket, 2014; Beck and Kenning, 2015; Jain and Bhatt, 2015; Stokes, 2015; Tanwar and Prasad, 2016; Wang, 2016; Carpentier et al., 2017; Saraswathy and Balakrishnan, 2017; Urbancova et al., 2017; Vorina et al., 2017; Akhir et al., 2018; Astrachan et al., 2018; Davies et al., 2018; Kaur et al., 2018; Nie et al., 2018; Okolo et al., 2018; Rubenstein et al., 2018; Sageder et al., 2018; Sharma and Prasad, 2018; Syed et al., 2018; Arasanmi and Krishna, 2019; Deepa and Baral, 2019; Dut̨u and Butucescu, 2019; Kamel, 2019; Madu et al., 2019; Qureshi, 2019; Tian and Robertson, 2019; West et al., 2019).

We postulate that the antecedent of employer branding, such as job design, competitive compensation, organizational environment, career progression, and employers’ prestige, causes to build employer brand image that eventually causes employees engagement and talent retention. The construct of employer brand image and employee engagement plays mediating role between antecedents of employer branding and talent retention. In the proposed conceptual framework, all relationships are rightly/positively causing their effects. It addresses novelty through the addition of job design as the antecedent of employer branding and the double mediation through employer brand image and employees’ engagement. On the basis of reviewed literature of employer brand image, employees’ engagement, and talent retention, it is conceptualized that competitive compensation (Chhabra and Sharma, 2014; Carpentier et al., 2017; Sharma and Prasad, 2018; Deepa and Baral, 2019), organizational environment (Jain and Bhatt, 2015; Stokes, 2015; Saraswathy and Balakrishnan, 2017; Urbancova et al., 2017; Kaur et al., 2018; Sageder et al., 2018; Sharma and Prasad, 2018; Deepa and Baral, 2019), career progression (Barrow and Mosley, 2007; Vaiman, 2008; Minchington, 2010; Priyadarshi, 2011; Truss et al., 2012; Dessler, 2016), organization’s prestige (Urde and Greyser, 2016; Carpentier et al., 2017; Saraswathy and Balakrishnan, 2017; Sharma and Prasad, 2018), and job design (Carpentier et al., 2017) have theoretical relationship with attitudinal variable employer brand image, which is theoretically linked with behavioral variable, i.e., mediator between attitude and consequence employees’ engagement (Kahn, 1990; Schaufeli et al., 2002; Inceoglu and Warr, 2012; Wang, 2016; Vorina et al., 2017), which has further theoretical relationship with behavioral consequences, talent retention at will (Arasanmi and Krishna, 2019; Qureshi, 2019; West et al., 2019). Hence, the final conceptual framework is depicted in Figure 2.

## Conclusion

The objective of the undertaken study was to develop an inclusive conceptual framework for the relationship between internal employer brand image and talent retention. For this purpose, authors have carried out an extensive and semi-systematic literature review and identified a number of antecedents and consequences that have been empirically established in numerous leading-edge research studies carried out around the globe at different time horizons in different prestigious indexing journals. The existing literature is reviewed using a topical approach, and 66 research studies, most recent from various repositories, were carefully chosen and reviewed based on the criteria. Such studies have been discerned and synthesized in order to establish a complete and accurate understanding of the phenomenon. Finally, a comprehensive and relatively rich conceptual framework has been proposed for future empirical explanations in various social settings via prospective research based on competitive compensation, organizational environment, career progression, job design, employer’s prestigious, internal employer brand image, employee’s engagement, and talent retention. During the research various constructs that arose through the literature review are acknowledgment of employees’ work, interaction with colleagues, colleague behavior, job environments, nature of the work, guidance, pay structure, promotion-related strategies and exercises, individual characteristics of employees, work-life balance, and economic condition, and so forth. These clues can also be used to forecast talent retention at will, though the link between said markers and talent retention will be contrary, as it is with turnover intentions. Analytical syntheses of these predecessors result in resemblances to antecedents of employer branding. As preceding literature established the process of antecedents leading to behavior and then to consequences employees’ engagement is taken into account in this research study as a behavior with several precursors that comprise the construct of employer branding; once employees are engrossed with their work, they will have a consequence in the form of willful employees’ retention. The suggested conceptual framework offers a roadmap for HR managers, HR consultants, and talent hunters and retainers to understand the workable link of employees’ positive attitude, its causes and effects on work and ultimately on the workplace. HR practitioners may add value to HR policies pertaining to hiring and retaining the talent. This may also help HR practitioners to confirm exemplary shifts from conventional human resource to the resource-based contemporary practices of human resource.

## Theoretical and Practical Contributions

The proposed conceptual framework adds theoretical value to the discourse of organizational behavior, HRM, and talent management. It offers a unique and novel addition of *Job Design* in the nexus between antecedents and consequences of employer branding. Moreover, the double mediating role of employer brand image and employees’ engagement adds theoretical value in the realm of knowledge. The proposed conceptual framework provides a roadmap for HR generalists, line managers, HR consultants, and talent hunters and retainers to understand the workable link of employees’ positive attitude, its causes, and effects on work and eventually on workplace. Practitioners may add value in HR policies pertaining to hiring and retaining the talent. This may also help HR practitioners to ensure the paradigmatic shift from traditional HR to the resource-based modern practices of HR.

## Limitations and Future Directions for Research

This probe appears to be limited in its scope of designing a conceptual framework based on literature gap and theoretical backdrop. Following theoretical underpinning particularly employer branding theory and theory of work design, the proposed conceptual framework merits empirical testing and explanation in different social settings in order to establish and increase its generalizability and to confirm or refute the theoretical relationship of the concepts. This framework contributes to theory and has implications for empirical research, theory, and practice, especially in policy and strategy design.

## Author Contributions

RA, JQ, and MA contributed to the conception and design of the study and wrote the first draft and sections. AH, VP, and NM revised and finalized the manuscript. AH and NM provided the scientific support during the research. All authors contributed to manuscript revision, read, and approved the submitted version.

## Conflict of Interest

The authors declare that the research was conducted in the absence of any commercial or financial relationships that could be construed as a potential conflict of interest.

## Publisher’s Note

All claims expressed in this article are solely those of the authors and do not necessarily represent those of their affiliated organizations, or those of the publisher, the editors and the reviewers. Any product that may be evaluated in this article, or claim that may be made by its manufacturer, is not guaranteed or endorsed by the publisher.
